# The Amygdala Excitatory/Inhibitory Balance in a Valproate-Induced Rat Autism Model

**DOI:** 10.1371/journal.pone.0055248

**Published:** 2013-01-29

**Authors:** Hui-Ching Lin, Po-Wu Gean, Chao-Chuan Wang, Yun-Han Chan, Po See Chen

**Affiliations:** 1 Department and Institute of Physiology, School of Medicine, National Yang-Ming University, Taipei, Taiwan; 2 Brain Research Center, National Yang-Ming University, Taipei, Taiwan; 3 Department of Pharmacology, College of Medicine, National Cheng Kung University, Tainan, Taiwan; 4 Department of Anatomy, College of Medicine, Kaohsiung Medical University, Kaohsiung, Taiwan; 5 Center for Drug Evaluation, Taipei, Taiwan; 6 Department of Psychiatry, National Cheng Kung University Hospital, College of Medicine, National Cheng Kung University, Tainan, Taiwan; 7 Addiction Research Center, National Cheng Kung University, Tainan, Taiwan; Kaohsiung Chang Gung Memorial Hospital, Taiwan

## Abstract

The amygdala is an important structure contributing to socio-emotional behavior. However, the role of the amygdala in autism remains inconclusive. In this study, we used the 28–35 days valproate (VPA)-induced rat model of autism to observe the autistic phenotypes and evaluate their synaptic characteristics in the lateral nucleus (LA) of the amygdala. The VPA-treated offspring demonstrated less social interaction, increased anxiety, enhanced fear learning and impaired fear memory extinction. Slice preparation and electrophysiological recordings of the amygdala showed significantly enhanced long-term potentiation (LTP) while stimulating the thalamic-amygdala pathway of the LA. In addition, the pair pulse facilitation (PPF) at 30- and 60-ms intervals decreased significantly. Whole-cell recordings of the LA pyramidal neurons showed an increased miniature excitatory postsynaptic current (EPSC) frequency and amplitude. The relative contributions of the AMPA receptor and NMDA receptor to the EPSCs did not differ significantly between groups. These results suggested that the enhancement of the presynaptic efficiency of excitatory synaptic transmission might be associated with hyperexcitibility and enhanced LTP in LA pyramidal neurons. Disruption of the synaptic excitatory/inhibitory (E/I) balance in the LA of VPA-treated rats might play certain roles in the development of behaviors in the rat that may be relevant to autism. Further experiments to demonstrate the direct link are warranted.

## Introduction

In humans, the amygdala is sensitive to environmental signs of emotional and social significance [1]–[2]. It could modulate emotional memory storage and drive autonomic response [3]. Emotional learning of social conventions through the amygdala may therefore contribute to socio-emotional regulation [4]–[7]. Moreover, social status has been found to be linked to the degree of amygdala activation [8]. A neuroimaging study found that the volume of the amygdala and cortical areas with amygdala connectivity correlates with the size and complexity of social networks in adult humans [9]. Researchers have shown that the amygdala responds preferentially to different social stimuli [10]–[11]. Within the amygdala, the laterally-situated nuclei (the lateral, lateral basal, mesial basal, and accessory basal nuclei) appear to contain neurons responsive to sensory social cues [1]–[2], [12]–[13]. However, most functional imaging studies reveal the amygdala function as a whole but do not distinguish the separate roles of specific nuclei in humans [14].

Fear and social signal processing could be impaired in the absence of a functional amygdala [15]. Dysfunction of the amygdala has been found to be related to disorders of fear processing, anxiety, and even social behaviors [16]–[18]. In recent years, there has been particular interest in the role of the amygdala in the development of autism spectrum disorder (ASD), a neurodevelopmental disorder with social deficit [19]–[22]. An amygdala theory of autism has been proposed after gaining an understanding of the neural basis of social intelligence [4], [23]. Previously, a role of the amygdala in determining the core social characteristics in ASD has been demonstrated on the basis of functional MR imaging [12], [24]. Altered amygdala activation in response to facial and emotion processing has also been noted in individuals with ASD [25]–[27]. In addition, postmortem studies of individuals with autism have shown cytoarchitectural and neuronal organization changes within the amygdala [28]–[29]. Structural MR imaging studies have also demonstrated abnormal amygdala volumes across multiple adolescents and adults with ASD [30]–[32]. Taken together, these results indicate that amygdala dysfunction may contribute to core social impairment in autism spectrum disorders [33].

Although human studies have suggested a role of the amygdala in the development of ASD, however, the role of amygdala in rodent models of autism has been less studied [34]. Among the rodent models, induction by exposure to the epigenectic modulator valproate (VPA) during the sensitive embryonic developmental stages has been established to mimic the gene–environment interacting property of autism [35]–[36]. The model is also of clinical significance because exposure to VPA during embryogenesis could cause developmental delays and ASD in humans if exposure occurs during the third week of gestation [36]–[40]. In the rodent model, a single prenatal injection of VPA on embryonic on day 11.5 disturbs enkephalinergic system functioning, the basal hedonic tone, and emotional responses [41].

Importantly, mature rats (3 months) in this model showed abnormal fear conditioning and processing in the lateral nucleus (LA) of the amygdala [42]. However, the acute amygdala slices used for *in vitro* electrophysiology in the previous study were prepared from rats at the early developmental stage (P12–P16). From that result, gaining an understanding of the possible links between the alteration of amygdala-associated behaviors and electrophysiological characteristics could be difficult, because the amygdala responses are tuned through the socialization process during development [10], [43]. In addition, the link between the amygdala and other brain regions changes with age [44], and age-related differences in the amygdala response to emotional cues have also been reported [45]. To further validate the role of the amygdala in the VPA-induced model at an early developmental age, in the current study, we used male 28–35 days VPA-induced model rats to observe both the amygdala-associated autistic phenotypes and the synaptic characteristics of the lateral nucleus (LA) of the amygdala by whole-cell patch clamp recording.

## Materials and Methods

### Valproate-induced model of autism

All procedures were approved by the Institutional Animal Care and Use Committee of the College of Medicine, National Cheng-Kung University (Tainan, Taiwan). Sprague Dawley rats were housed four to a cage in a temperature-controlled (24°C) animal colony under a 12∶12-h light/dark cycle, with lights on at 7:00 AM. All behavioral procedures took place during the light cycle. Rats were mated, with pregnancy determined by the presence of a vaginal plug on embryonic day 1 (E1). The sodium salt of valproic acid (NaVPA, Sigma-Aldrich) was dissolved in 0.9% saline to obtain a concentration of 150 mg/mL, pH 7.3. The dosing volume was 3.3 mL/kg, with the dosage adjusted according to the body weight of the dam on the day of injection. Treated rats received a single intraperitonal injection of 500 mg/kg NaVPA and control dams a single injection of saline on E12.5 [42]. Rats were housed individually and were allowed to raise their own litters until weaning. The offspring were then separated and housed in cages of 3–4 rats until the behavioral experiments. Both behavioral and electrophysiological experiments were conducted on P28–35.

### Behavioral testing

Behavioral experiments of fear conditioning and extinction were performed in an operant chamber (Med Associates, St Albons, VT). The behavioral experiment examining social interaction and the open field test were performed in a white plastic box. The behavioral experiment of the elevated plus maze was performed using a platform in the shape of a cross. Video tracking software (Ethovision, Noldus, The Netherlands) was used for automatic recording and analysis. A total of 28 saline-treated and 37 VPA-treated rats from 20 litters were sacrificed for behavioral tests. The open field (28 saline-treated, 37 VPA-treated), elevated plus maze (27 saline-treated, 34 VPA-treated) and social interaction (26 saline-treated, 36 VPA-treated) tests were performed continuously. All rats were weight-matched and aged-matched. The rats that did not move for over 10 minutes in the open field were excluded in the open field or social interaction tests. For the first 11 saline-treated and 10 VPA-treated rats, the fear conditioning and extinction tests were performed after the social interaction tests.

### Social interaction

Rats were separated and housed individually the night before the experiment. The apparatus was a white plastic box (50×40×40 cm). Rats were matched in terms of gender and weight. After a 60-min habituation period in the room, one VPA-treated and one control rat were placed into the apparatus over a period of 20 minutes. The percentage of time spent following, mounting, grooming each other, and sniffing of any body part were taken as indicators of social engagement [46].

### Open field test

The rats were inserted for 15 minutes in a white plastic box (50×40×40 cm). The percentage of time spent in the central zone (25% of the surface area) and the total distance (cm) moved were measured.

### Elevated plus-maze

The rats were inserted for 5 minutes in a standard elevated plus-maze, which consisted of two opposite open arms and two opposite closed arms (50×5×40 cm) arranged at right angles. The percentage of time spent in each arm, the total distance (cm) moved and the velocity (cm/s) were measured.

### Fear conditioning

Fear conditioning occurred in a 32×26×26 cm operant chamber (Med Associates, St Albons, VT) which controlled by FreezeScan software (Clever Systems, Reston, VA). The shock floor consisting of stainless-steel rods was wired to a shock generator for foot shock delivery. The house light provided illumination during all sessions. The chamber was cleaned with 75% ethanol before each rat was trained or tested for contextual fear conditioning. On the first day of training, the rats were transported in their home cage to a behavioral room. After a 60-min habituation period in the room, the rats were placed in the training chamber for 180 s. After the acclimation period, the rats were presented with a pure tone (15 s, 1 kHz, 80 dB) that coterminated with a foot shock (1 s, 0.8 mA). This tone–foot shock pairing procedure was repeated three times with an inter-trial interval of 60 s. After the last tone paired with shock delivery, the rats were allowed to explore the context for 2 min before removal from the chamber. At 24 h after training, the rats were returned to the training chamber for 5 min without exposure to the tone or the foot shock as a contextual fear test. At the end of the contextual test, the rats were returned to their home cage. Approximately 1 h later, the rats were placed in a novel context (an opaque Plexiglas box) for a 180 s baseline period followed by a pure tone (15 s, 1 kHz, 80 dB) repeated ten times with an inter-trial interval of 30 s to assess the extinction of fear conditioning. The grid floor was replaced with a smooth Plexiglas floor and the chamber was cleaned with 1% acetic acid before each rat was tested. Freezing was defined as the absence of any movement except respiration and was measured automatically using FreezeScan software.

### Brain slice preparation and electrophysiological recordings of the amygdala

After demonstrating the enhanced acquisition and impaired extinction of fear memory in VPA-treated rats, we evaluated the synapse characteristics of the amygdala on the electrophysiological level to check for possible alterations. Slice preparation and electrophysiological recordings of the amygdala were performed for the male 28–35 days VPA-induced model rats. Brain slices were prepared as described previously [47]. Evoked EPSCs were created by electrical stimulation of the external capsule (EC), which contained fibers from the auditory cortex to the LA, with a concentric bipolar stimulating electrode. Electrical stimuli (150 µs in duration) were delivered at a frequency of .05 Hz. Baseline field potentials were adjusted to 30–40% of the maximal responses. Long term potentiation (LTP) was elicited by pairing pre-synaptic stimulation (2 Hz, 200 pulses) with post-synaptic depolarization to −5 mV as described previously [48]. Bicuculline (2 µM) was present in the perfusion solution. To survey the attribution of enhanced synapse strength, whole-cell recordings were made from the soma of visually-identified pyramidal-like neurons located in the LA. Neurons were identified as projection neurons based on their morphology in cesium methanesulfonate-containing electrodes. In potassium gluconate-containing electrodes, these neurons were also identified by their intrinsic electrophysiological properties [49].

To avoid the possible confounding effect of behavioral tests, an additional 15 saline-treated and 26 VPA-treated rats from 12 litters were sacrificed for electrophysiological recordings. Among them, 6 saline-treated and 6 VPA-treated rats were sacrificed for the long term potentiation (LTP) protocol. The remaining 9 saline-treated and 20 VPA-treated rats were sacrificed for AMPA/NMDA ratio (9 neurons from 9 saline-treated rats, 12 neurons from 12 VPA-treated rats), PPF ratio (10 neurons from 9 saline-treated rats, 15 neurons from 15 VPA-treated rats) and mEPSCs evaluation (13 neurons from 9 saline-treated rats, 9 neurons from 9 VPA-treated rats).

### Statistical analysis

We analyzed the data using the statistical package for social sciences (SPSS 12). Categorical variables were expressed in numbers and percentages, and continuous variables as the mean±SD unless otherwise specified. The variables were assessed using the chi-square test, Student's t-test, and one-way ANOVA. If the sample size was less than 25, we used the Wilcoxon Rank Sum test or the Mann-Whitney U test for continuous variables. The difference between groups was considered significant if the p value<0.05.

## Results

According to the design of the experimental regimen, the pregnant female rats had received saline or VPA on E12.5. On postnatal day 28–35 d, their male offspring were tested using serial behavioral tasks, including the social interaction (SI), open field (OF) and elevated plus (EPM) maze tasks, in one day. The day after, the same offspring either underwent the fear condition test or were sacrificed for electrophysiological recordings.

### Decreased social interaction and enhanced anxiety behavior at the early developmental stage

**Figure 1:**
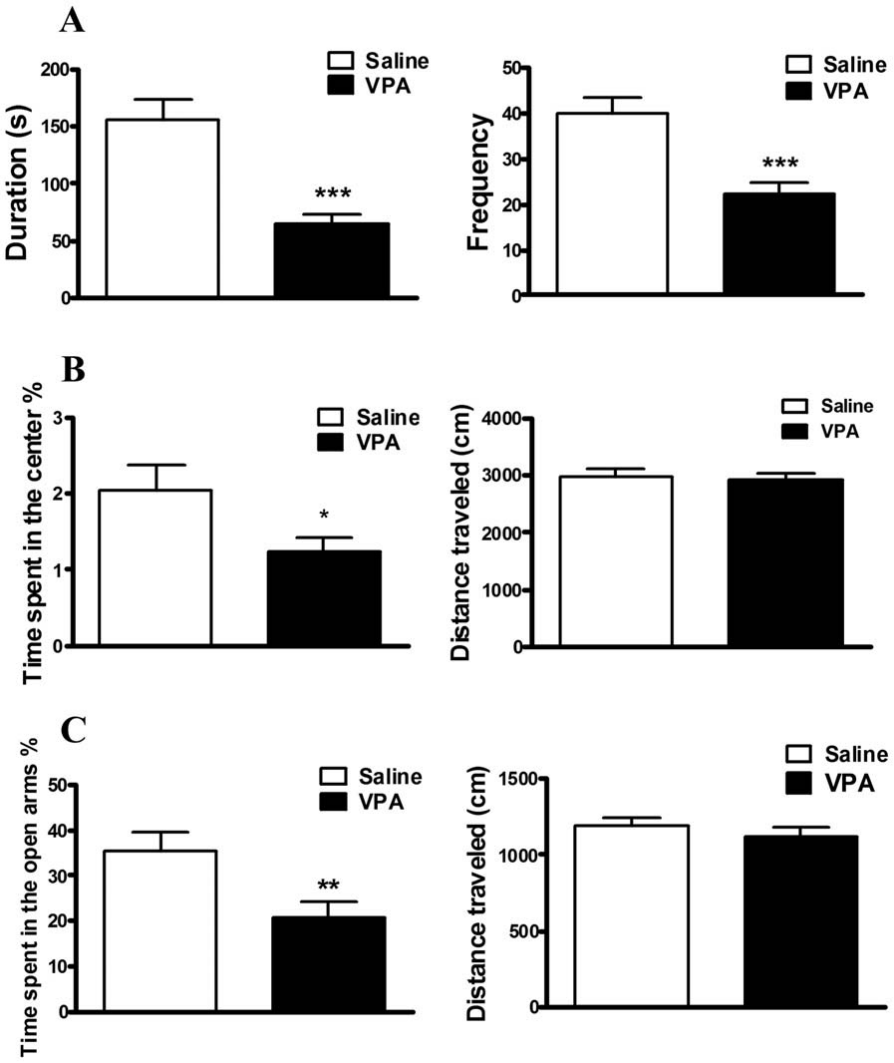
Social interaction and anxiety behavior in the male VPA offspring. Social impairment in (A) the social interaction test. The male VPA offspring exhibited a lower duration and frequency compared with the male saline offspring in the social interaction test. ***p<0.001 (Saline, n = 26; VPA, n = 36). Increased anxiety in (B) the open field test. The male VPA offspring spent less time in the center percentage, but there was no significant difference in the distance traveled as compared with the male saline offspring. *p<0.05 (Saline, n = 28; VPA, n = 37). (C) Elevated plus maze test. The male VPA offspring spent a lower percentage of time in the open arms, but there was no significant difference in the distance traveled as compared with the saline offspring. **p<0.01 (Saline, n = 27; VPA, n = 34).

Herein, we measured the social behavior characteristics of the 28–35-day-old male VPA-treated rats and also evaluated behaviors that likely involve amygdala processing. Figure 1A shows that the duration of social interaction was 64.89±45.22 (n = 36) and 156.0±88.84 (n = 26) in the VPA-treated and control rats, respectively. The frequency of social interaction was 22.44±14.54 (n = 36) and 40.62±17.67 (n = 26) in the VPA-treated and control rats, respectively. The results of the social interaction test showed that the male VPA-treated rats exhibited a significantly lower social interaction duration and frequency than the male saline-treated rats (duration: t_(60)_ = 5.29, *p*<0.001; frequency: t_(60)_ = 4.44, *p*<0.001) (Figure 1A). The results indicated that the VPA-treated rat model presented social, anxiety and fear behaviors that may be relevant to some of the behavioral symptoms exhibited in individuals with ASD. Because the core symptoms of autism are frequently accompanied by aberrant anxiety behavior, we then examined whether the male VPA-treated rats also presented increased amygdala-associated anxiety behavior at this developmental stage. In the open field test, we found that the male VPA-treated rats spent significantly less time in the center percentage (saline-treated group: 2.05±1.74 (n = 28); VPA-treated group: 1.25±1.06 (n = 37); t_(63)_ = 2.31, p = 0.02), but the total distance traveled did not differ (saline-treated group: 2967.68±801.27 (n = 28); VPA-treated group: 2917.87±735.33 (n = 37); t_(63)_ = 0.26, p = 0.80) (Figure 1B). In the elevated plus maze test, the male VPA-treated rats also spent significantly less time in the open arms (saline-treated group: 35.28±22.61 (n = 27); VPA-treated group: 20.79±19.88 (n = 34); t_(59)_ = 2.66, p = 0.01), with no difference in the total distance traveled (saline-treated group: 1197.08±255.92 (n = 27); VPA-treated group: 1124.56±346.21 (n = 34); t_(59)_ = 0.91, p = 0.37) (Figure 1C).

### Abnormal fear conditioning and enhanced LTP at the amygdala synapse

**Figure 2:**
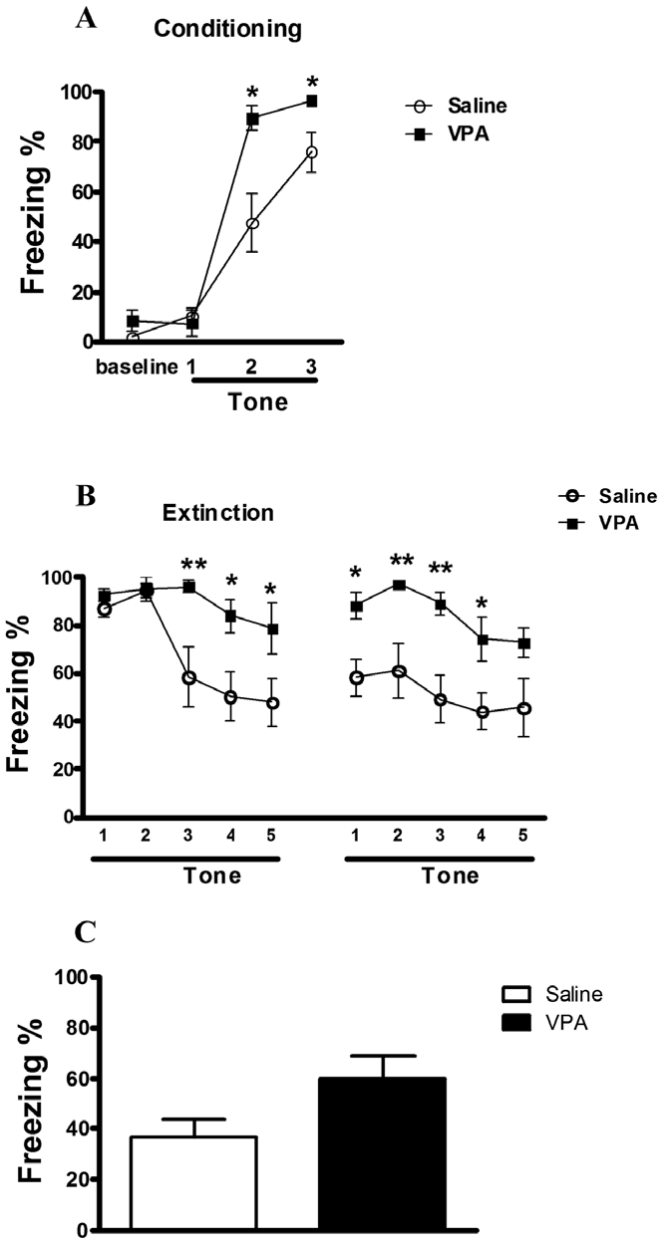
Impaired fear memory formation and extinction in the male VPA offspring. (A) Freezing behavior on the conditioning day. Data are the 180-s averages for the period before (baseline) and after each of the 3 tone–shock conditioning trials. (B) Freezing behavior during the extinction session, which occurred 24 h after conditioning. One block is the mean of two CS trials. (C) Freezing behavior during the 5-min contextual test. *p<0.05, **p<0.01 (Saline, n = 11; VPA, n = 10).

The above results were in accordance with the behavioral symptoms exhibited in individuals with ASD, who present a high anxious reaction [50]. The over-anxious response could be caused by alteration in amygdala activity. Therefore, we used the fear-conditioning paradigms to further assess the amygdala-associated fear memory and emotional response of the saline-treated group (n = 11) and VPA-induced model (n = 10) at this age [42], [51]. We first determined whether VPA-treated rats presented changes in fear memory formation using cued and contextual fear-conditioning paradigms. On the conditioning day, two-way ANOVA revealed a significant interaction between groups [*F*(3, 57) = 5.85, p = 0.001] (Figure 2). Retention of memory was tested 24 h after training. Rats were tested in terms of their fear to the cue by assessing the freezing behavior on two successive days. For the extinction session, rats were placed in a novel context and presented with ten auditory cues. As shown in Figure 2, the cue-induced freezing was comparable between groups. During the extinction session, group interaction emerged on day 1 [*F*(4, 76) = 3.55, p = 0.01] and group interaction emerged in day 2 [*F*(4, 76) = 0.25, p = 0.91] in day 2. After that, the rats were returned to the training chamber for 5 min without exposure to the tone or foot shock for a context fear test. The contextual freezing responses of the VPA-treated rats were more obvious than those of the controls, whereas no difference in freezing was found between groups (saline-treated group: 36.96±21.22 (n = 11); VPA-treated group: 59.52±30.08 (n = 10); Mann-Whitney U test, p = 0.07) (Figure 2C).

### The synaptic characteristics of the pyramidal neurons in the amygdala

**Figure 3:**
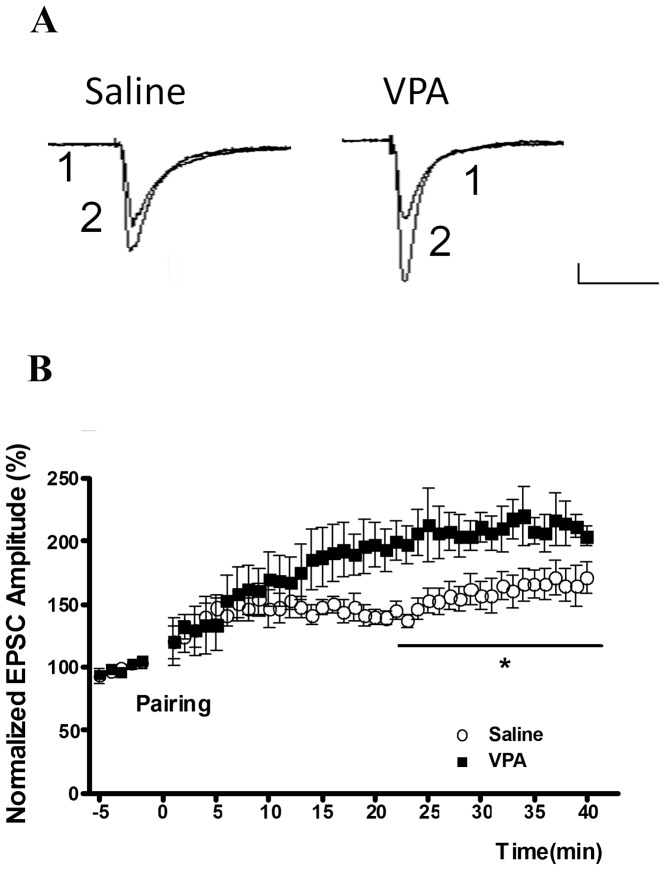
The LTP is enhanced in the VPA offspring at the thalamic–amygdala synapses. (A) Sample traces are the averaged EPSCs taken before (1) and 40 min after (2) LTP induction in the saline (n = 6) and VPA (n = 6) offspring. Scale; 50 ms, 40 pA. (B) LTP in the LA neurons was induced by pairing pre-synaptic stimulation (2 Hz, 200 pulses) with post-synaptic depolarization to −5 mV. The amplitude of individual EPSCs was normalized to the averaged amplitude of EPSCs during the 5-min baseline recordings just before LTP induction. *p<0.05; scale 50 ms, 40 pA.

LTP while stimulating the thalamic–amygdala pathway in LA neurons was induced by pairing pre-synaptic stimulation (2 Hz, 200 pulses) with post-synaptic depolarization to −5 mV as described previously (Yu et al., 2008). The amplitude of the individual excitatory postsynaptic current (EPSC) was normalized to the averaged amplitude of the EPSCs during the 5 min baseline recordings just before LTP. Two-way ANOVA revealed a significant interaction between groups [*F*(39, 390) = 2.43, p = 0.0001] We also observed that the LTP was significantly enhanced in the VPA-treated rats at the thalamic–amygdala synapses (p<0.5) (Figure 3).

**Figure 4:**
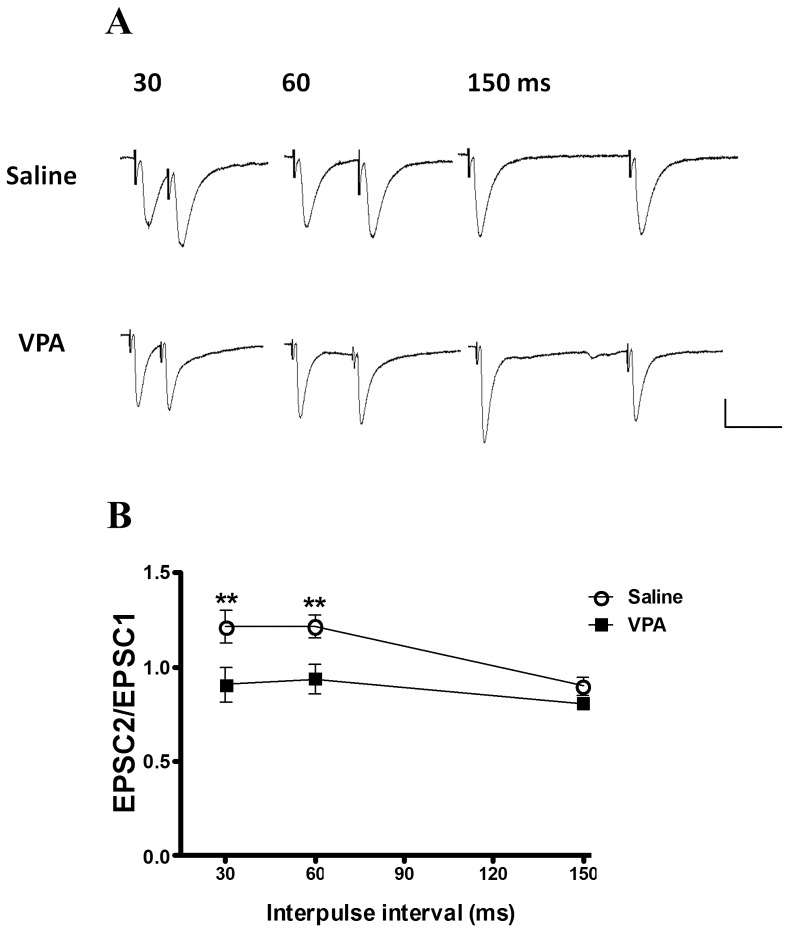
Paired-pulse facilitation (PPF) in the LA neurons of the VPA offspring. (A) Sample traces of PPF in the saline (n = 10) and VPA (n = 15) offspring. (B) Plot of PPF for 30-, 60-, and 150-ms interpulse intervals at the thalamic–amygdala synapse. **p<0.01; scale 50 ms, 40 pA.

To determine whether the increased synaptic strength recorded in the VPA-treated rats involved a presynaptic mechanism, we analyzed the pair pulse facilitation (PPF) in slices from the VPA-treated and control rats [52]–[53]. The ratio of the amplitude of the second EPSC to the amplitude of the first EPSC was examined at different interpulse intervals. In the thalamic–amygdala pathway, the PPF at 30- and 60-ms intervals in the VPA-treated rats (n = 15) was significantly lower than that of the controls (n = 10) (30 ms: 0.86±0.30 in VPA-treated rats, 1.21±0.28 in controls, Mann-Whitney U test, p = 0.003; 60 ms: 0.90±0.27 in VPA-treated rats, 1.21±0.19 in controls, Mann-Whitney U test, p = 0.002) (Figure 4). This result suggested that enhanced synaptic efficacy after fear conditioning is mediated at least in part by an increase in the presynaptic release probability.

**Figure 5:**
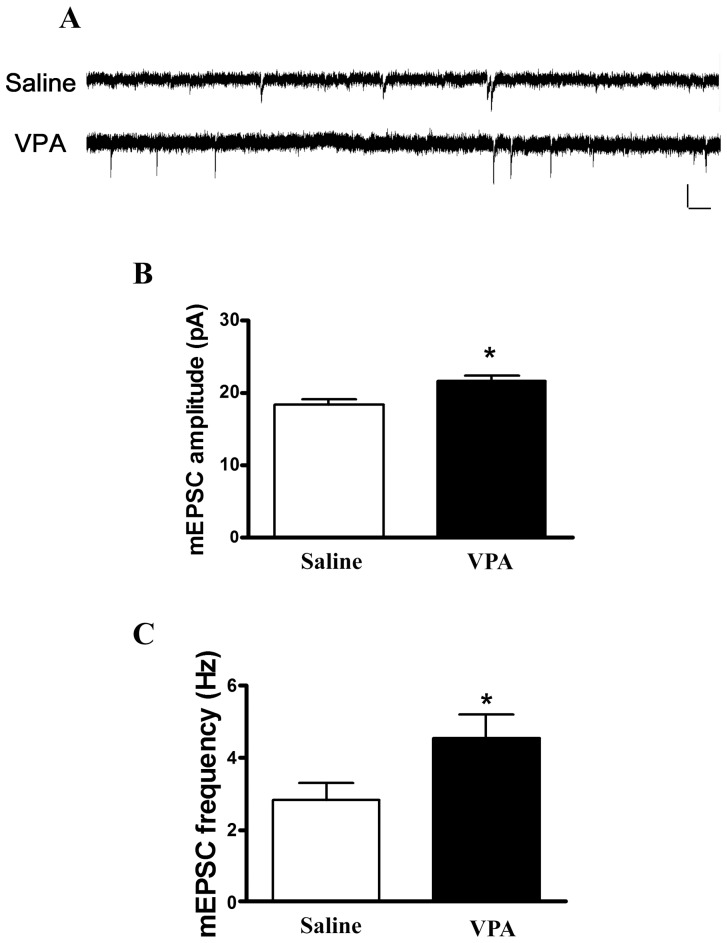
Miniature excitatory postsynaptic currents (mEPSCs) in the LA neurons. (A) Sample traces of mEPSCs taken from slices of saline (n = 13) and VPA offspring (n = 9). The mEPSCs were recorded in the LA neurons at a holding potential of −70 mV in the presence of bicuculline (10 µM) and TTX (0.5 µM) (B and C). Plot average of (B) the amplitude and (C) the frequency of mEPSCs in the saline and VPA offspring. *p<0.05; scale 200 ms, 50 pA.

**Figure 6:**
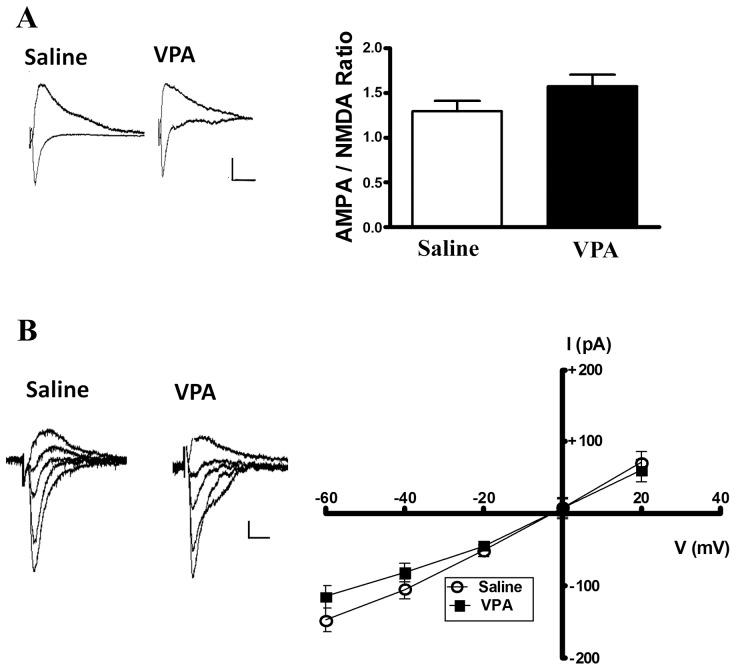
The AMPA/NMDA ratio in the LA neurons. (A) Sample traces showing the synaptic current at −70 and +40 mV in the saline (n = 9) and VPA (n = 12) offspring. Average AMPA/NMDA ratio in the saline and VPA offspring at the thalamic–amygdala pathway. Scale; 50 ms, 40 pA. (B) No difference was observed in the current–voltage curve of AMPA receptor-mediated EPSCs in the LA neurons from the saline and VPA offspring. A sample trace and plot of the I–V curve of AMPA-mediated EPSCs were recorded in the presence of APV (20 µM) and bicuculline (10 µM) at holding potentials of −60 mV to +20 mV in the saline (n = 7) and VPA offspring (n = 8) at the thalamic–amygdala pathway. Scale; 50 ms, 40 pA.

We then examined whether excitatory synaptic transmission was altered in the VPA-treated rats by recording the miniature excitatory post-synaptic current (mEPSCs). The recorded mEPSCs in the presence of bicuculline (10 µM) and TTX (0.5 µM) revealed a significantly higher frequency (4.77±1.85 Hz, n = 9 in the VPA-treated group; 2.67±1.63 Hz, n = 13 in the saline-treated group) and amplitude (21.59±2.09 pA, n = 9 in the VPA-treated group; 18.25±3.19 pA, n = 13 in the saline-treated group) in slices from the male VPA-treated rats (frequency: Mann-Whitney U test, p = 0.04); amplitude: Mann-Whitney U test, p = 0.01) (Figure 5). Furthermore, we measured the relative contributions of the AMPA receptor and NMDA receptor to the EPSCs [54]–[55]. The AMPA EPSC was evoked when the neurons were voltage-clamped at −70 mV, whereas the NMDA EPSC was determined as the current amplitude at 50 ms after the peak EPSC amplitude at a holding potential of +40 mV [56]. In the thalamic–amygdala pathway, the AMPA/NMDA ratios were 1.02±0.46 (n = 12) and 0.95±0.37 (n = 9) in the VPA-treated and control rats, respectively. The ratio was not significantly different between groups (Mann-Whitney U test, p = 0.69, Figure 6A). We also determined the AMPA-mediated I–V curve in the presence of bicuculline (10 µM) and APV (20 µM). Consistent with the above result, the AMPA-mediated I–V curve did not differ significantly between groups [*F*(4, 65) = 1.28, p = 0.29] (Figure 6B).

## Discussion

### Validation of the VPA-induced model at an early developmental age

Previous studies have demonstrated that mature adult offspring of VPA-treated rats show less social interaction [40]. Moreover, this model shows sexual dimorphism characteristics for the measured behavior [57]. However, the behavioral pattern at an early age has not yet been investigated. In this study, our behavioral experiment results first showed the behavior characteristics of the VPA model rats at an early developmental age. Although a previous study demonstrated that mature VPA model rats showed similar behavior [42], however, it is of importance to further identify the developmental and gender effects while measuring behavior patterns. Particularly, certain behavioral changes of the offspring of the VPA-treated rats are likely related to amygdala function, and the amygdala responses could be tuned through the social stress process during development [10], [43]. In addition, the gender effect might also play an important role in the behavioral changes related to the amygdala function [58]–[59]. It is necessary to test the female VPA-induced model rats at various development stages to observe the amygdala-associated autistic phenotypes and evaluate their synaptic characteristics in further studies.

### The roles of amygdala-associated behaviors in the VPA-induced model

The alteration of amygdala-associated behaviors observed herein provided further evidence that dysfunction of the amygdala could be related to the core symptoms presented in autism at an early developmental age [19]–[22]. As we were particularly interested in the role of the amygdala in autism, we further measured the electrophysiological features of the LA in the VPA-treated rats. Because plasticity in the LA is known to be associated with the learning and extinction of fear memory, we therefore first tested whether the LTP had changed in the LA of the VPA-treated rats. The results indicated that the LTP in the LA was significantly enhanced in the VPA-treated rats at the thalamic–amygdala synapses. Whether the electrophysiological activity correlates with the enhanced amygdala activity revealed in autistic subjects merits further investigation [60]–[61].

### The amygdala E/I balance and autistic phenotypes

Interestingly, enhanced plasticity has also been noted in other brain areas of VPA-treated rats [35], [42], [62]. Moreover, the VPA model rats showed a significant enhancement of the local recurrent connectivity formed by neocortical pyramidal neurons in the cortex [35], and the excitatory connections were noted to be more plastic, displaying enhanced long-term potentiation of the strength of the synapses [35]. Various rodent models of autism have revealed that the increased ratio of synaptic excitation/inhibition (E/I) in key neural circuits might play certain roles in the pathophysiology of autism spectrum disorders [63]–[65]. Furthermore, both genetic and non-genetic factors cause the imbalanced E/I balance [63]. Previously, the VPA-induced model revealed impairment in the neocortical pyramidal neuronal intrinsic excitability and an increase in the NMDA synaptic currents [35]. Importantly, both electrophysiological abnormalities were developmental stage-dependent [66]. In the amygdala, the pyramidal neurons of the model rats showed a deficit in inhibition [42], and disruption of the inhibitory circuits has been considered as one cause of certain autistic phenotypes in the model [67]. In the current study, we demonstrated that the presynaptic efficiency of excitatory synaptic transmission might be associated with hyperexcitibility and enhanced LTP in LA pyramidal neurons. Whether or not disruption of the synaptic E/I balance in the LA of VPA-treated rats causes autistic phenotypes merits further investigation.

### The molecular mechanism

How prenatal VPA treatment alters the presynaptic efficiency of excitatory synaptic transmission in the amygdala remains unclear. A study showed the selective impact of histone deacetylases on the dynamics of evoked excitatory neurotransmission in a model of Rett's Disorder, a pervasive developmental disorder with some features that are clinically similar to those seen in ASD [68]. Prenatal VPA exposure in the current model could cause a transient increase in acetylated histone levels in the embryonic brain [59]. These findings together suggested that VPA-induced histone hyperacetylation may play a key role in evoking excitatory neurotransmission. Our previous in vitro study demonstrated that VPA treatment could regulate the gene levels of two post-synaptic cell adhesion molecules (CAM) (neuroligin-1 and neuregulin-1) and two extracellular matrix (ECM) proteins (neuronal pentraxin-1 and thrombospondin-3) in a primary astrocyte culture in a time- and concentration-dependent manner. Moreover, sodium butyrate, another histone deacetylase inhibitor, but not glycogen synthase kinase-3 beta inhibitors, could mimic these effects. The constitutions of CAM and ECM play important roles in the synaptic E/I balance and developmental brain disorders. For example, the pair neuroligins (NL) and beta-neurexin are emerging as central organizing molecules for excitatory glutamatergic and inhibitory GABAergic synapses in the mammalian brain [69]–[71], and NL-1 has been noted to regulate synaptic plasticity in the amygdala [72]. In humans, alterations in genes encoding CAMs and ECMs have recently been implicated in autism and developmental brain disorders [73]–[75]. As the next step in our research, we would like to test the possible associations in this model in vivo.

## Conclusions

The amygdala has been implicated as a key component of the social cognitive circuitry. In the VPA-induced autism model, our results further indicated that the enhancement of presynaptic efficiency of excitatory synaptic transmission might be associated with hyperexcitibility and enhanced LTP in LA pyramidal neurons at an early developmental age, and the increased ratio of synaptic excitation/inhibition (E/I) in the amygdala might be associated with the characteristic behavior in this model. Identifying factors affecting the E/I balance in the amygdala could give us the opportunity to prevent and treat related neurodevelopment disorders. However, interpretation is limited without further direct evidence. Further experiments including direct recordings from amygdala neurons during the behavioral experiment and observing the behavioral consequences after modulating the amygdala E/I balance could give us the opportunity to demonstrate the link between the two.

## References

[1] Adolphs R, Spezio M (2006) Role of the amygdala in processing visual social stimuli. Prog Brain Res 156: 363–378. 17015091 10.1016/S0079-6123(06)56020-0

[2] Kuzmanovic B, Bente G, von Cramon DY, Schilbach L, Tittgemeyer M, et al (2011) Imaging first impressions: Distinct neural processing of verbal and nonverbal social information. Neuroimage 60: 179–188. 22227133 10.1016/j.neuroimage.2011.12.046

[3] LeDoux J (2003) The emotional brain, fear, and the amygdala. Cell Mol Neurobiol 23: 727–738. 14514027 10.1023/A:1025048802629 PMC11530156

[4] Amaral DG (2002) The primate amygdala and the neurobiology of social behavior: implications for understanding social anxiety. Biol Psychiatry 51: 11–17. 11801227 10.1016/s0006-3223(01)01307-5

[5] Emery NJ, Capitanio JP, Mason WA, Machado CJ, Mendoza SP, et al (2001) The effects of bilateral lesions of the amygdala on dyadic social interactions in rhesus monkeys (Macaca mulatta). Behav Neurosci 115: 515–544. 11439444

[6] LeDoux JE (1995) Emotion: clues from the brain. Annu Rev Psychol 46: 209–235. 7872730 10.1146/annurev.ps.46.020195.001233

[7] Markowitsch HJ, Staniloiu A (2011) Amygdala in action: relaying biological and social significance to autobiographical memory. Neuropsychologia 49: 718–733. 20933525 10.1016/j.neuropsychologia.2010.10.007

[8] Muscatell KA, Morelli SA, Falk EB, Way BM, Pfeifer JH, et al (2012) Social status modulates neural activity in the mentalizing network. Neuroimage In press. 10.1016/j.neuroimage.2012.01.080 PMC3909703 22289808

[9] Bickart KC, Wright CI, Dautoff RJ, Dickerson BC, Barrett LF (2011) Amygdala volume and social network size in humans. Nat Neurosci 14: 163–164. 21186358 10.1038/nn.2724 PMC3079404

[10] Lederbogen F, Kirsch P, Haddad L, Streit F, Tost H, et al (2011) City living and urban upbringing affect neural social stress processing in humans. Nature 474: 498–501. 21697947 10.1038/nature10190

[11] Pejic T, Hermann A, Vaitl D, Stark R (2011) Social anxiety modulates amygdala activation during social conditioning. Social Cognitive and Affective Neuroscience In press. 10.1093/scan/nsr095 PMC3594720 22198970

[12] Baron-Cohen S, Ring HA, Bullmore ET, Wheelwright S, Ashwin C, et al (2000) The amygdala theory of autism. Neurosci Biobehav Rev 24: 355–364. 10781695 10.1016/s0149-7634(00)00011-7

[13] Gadziola MA, Grimsley JM, Shanbhag SJ, Wenstrup JJ (2012) A novel coding mechanism for social vocalizations in the lateral amygdala. J Neurophysiol 107: 1047–1057. 22090463 10.1152/jn.00422.2011 PMC3289453

[14] Saygin ZM, Osher DE, Augustinack J, Fischl B, Gabrieli JD (2011) Connectivity-based segmentation of human amygdala nuclei using probabilistic tractography. Neuroimage 56: 1353–1361. 21396459 10.1016/j.neuroimage.2011.03.006 PMC3102511

[15] Becker B, Mihov Y, Scheele D, Kendrick KM, Feinstein JS, et al (2012) Fear Processing and Social Networking in the Absence of a Functional Amygdala. Biol Psychiatry 72: 70–77. 22218285 10.1016/j.biopsych.2011.11.024

[16] Blair RJ, Peschardt KS, Budhani S, Mitchell DG, Pine DS (2006) The development of psychopathy. J Child Psychol Psychiatry 47: 262–276. 16492259 10.1111/j.1469-7610.2006.01596.x

[17] Cottraux J (2005) Recent developments in research and treatment for social phobia (social anxiety disorder). Curr Opin Psychiatry 18: 51–54. 16639184

[18] Damsa C, Maris S, Pull CB (2005) New fields of research in posttraumatic stress disorder: brain imaging. Curr Opin Psychiatry 18: 55–64. 16639185

[19] Adolphs R (2009) The social brain: neural basis of social knowledge. Annu Rev Psychol 60: 693–716. 18771388 10.1146/annurev.psych.60.110707.163514 PMC2588649

[20] Hampton AN, Adolphs R, Tyszka MJ, O'Doherty JP (2007) Contributions of the amygdala to reward expectancy and choice signals in human prefrontal cortex. Neuron 55: 545–555. 17698008 10.1016/j.neuron.2007.07.022

[21] Kennedy DP, Glascher J, Tyszka JM, Adolphs R (2009) Personal space regulation by the human amygdala. Nat Neurosci 12: 1226–1227. 19718035 10.1038/nn.2381 PMC2753689

[22] Tsuchiya N, Kawasaki H, Oya H, Howard MA 3rd, Adolphs R (2008) Decoding face information in time, frequency and space from direct intracranial recordings of the human brain. PLoS One 3: e3892. 19065268 10.1371/journal.pone.0003892 PMC2588533

[23] Heritch AJ, Henderson K, Westfall TC (1990) Effects of social isolation on brain catecholamines and forced swimming in rats: prevention by antidepressant treatment. J Psychiatr Res 24: 251–258. 2266513 10.1016/0022-3956(90)90014-h

[24] Baron-Cohen S, Ring HA, Wheelwright S, Bullmore ET, Brammer MJ, et al (1999) Social intelligence in the normal and autistic brain: an fMRI study. Eur J Neurosci 11: 1891–1898. 10336657 10.1046/j.1460-9568.1999.00621.x

[25] Dalton KM, Nacewicz BM, Johnstone T, Schaefer HS, Gernsbacher MA, et al (2005) Gaze fixation and the neural circuitry of face processing in autism. Nat Neurosci 8: 519–526. 15750588 10.1038/nn1421 PMC4337787

[26] Hadjikhani N, Joseph RM, Snyder J, Tager-Flusberg H (2007) Abnormal activation of the social brain during face perception in autism. Hum Brain Mapp 28: 441–449. 17133386 10.1002/hbm.20283 PMC6871469

[27] Wang AT, Dapretto M, Hariri AR, Sigman M, Bookheimer SY (2004) Neural correlates of facial affect processing in children and adolescents with autism spectrum disorder. J Am Acad Child Adolesc Psychiatry 43: 481–490. 15187809 10.1097/00004583-200404000-00015

[28] Bailey A, Luthert P, Dean A, Harding B, Janota I, et al (1998) A clinicopathological study of autism. Brain 121 Pt 5: 889–905. 9619192 10.1093/brain/121.5.889

[29] Schumann CM, Amaral DG (2006) Stereological analysis of amygdala neuron number in autism. J Neurosci 26: 7674–7679. 16855095 10.1523/JNEUROSCI.1285-06.2006 PMC6674270

[30] Aylward EH, Minshew NJ, Goldstein G, Honeycutt NA, Augustine AM, et al (1999) MRI volumes of amygdala and hippocampus in non-mentally retarded autistic adolescents and adults. Neurology 53: 2145–2150. 10599796 10.1212/wnl.53.9.2145

[31] Nacewicz BM, Dalton KM, Johnstone T, Long MT, McAuliff EM, et al (2006) Amygdala volume and nonverbal social impairment in adolescent and adult males with autism. Arch Gen Psychiatry 63: 1417–1428. 17146016 10.1001/archpsyc.63.12.1417 PMC4767012

[32] Rojas DC, Smith JA, Benkers TL, Camou SL, Reite ML, et al (2004) Hippocampus and amygdala volumes in parents of children with autistic disorder. Am J Psychiatry 161: 2038–2044. 15514404 10.1176/appi.ajp.161.11.2038

[33] Todd RM, Anderson AK (2009) Six degrees of separation: the amygdala regulates social behavior and perception. Nat Neurosci 12: 1217–1218. 19783979 10.1038/nn1009-1217

[34] Silverman JL, Yang M, Lord C, Crawley JN (2010) Behavioural phenotyping assays for mouse models of autism. Nat Rev Neurosci 11: 490–502. 20559336 10.1038/nrn2851 PMC3087436

[35] Rinaldi T, Silberberg G, Markram H (2008) Hyperconnectivity of local neocortical microcircuitry induced by prenatal exposure to valproic acid. Cereb Cortex 18: 763–770. 17638926 10.1093/cercor/bhm117

[36] Wagner GC, Reuhl KR, Cheh M, McRae P, Halladay AK (2006) A new neurobehavioral model of autism in mice: pre- and postnatal exposure to sodium valproate. J Autism Dev Disord 36: 779–793. 16609825 10.1007/s10803-006-0117-y

[37] Genton P, Semah F, Trinka E (2006) Valproic acid in epilepsy: pregnancy-related issues. Drug Saf 29: 1–21. 16454531 10.2165/00002018-200629010-00001

[38] Miyazaki K, Narita N, Narita M (2005) Maternal administration of thalidomide or valproic acid causes abnormal serotonergic neurons in the offspring: implication for pathogenesis of autism. Int J Dev Neurosci 23: 287–297. 15749253 10.1016/j.ijdevneu.2004.05.004

[39] Rasalam AD, Hailey H, Williams JH, Moore SJ, Turnpenny PD, et al (2005) Characteristics of fetal anticonvulsant syndrome associated autistic disorder. Dev Med Child Neurol 47: 551–555. 16108456 10.1017/s0012162205001076

[40] Schneider T, Przewlocki R (2005) Behavioral alterations in rats prenatally exposed to valproic acid: animal model of autism. Neuropsychopharmacology 30: 80–89. 15238991 10.1038/sj.npp.1300518

[41] Schneider T, Ziolkowska B, Gieryk A, Tyminska A, Przewlocki R (2007) Prenatal exposure to valproic acid disturbs the enkephalinergic system functioning, basal hedonic tone, and emotional responses in an animal model of autism. Psychopharmacology (Berl) 193: 547–555. 17497229 10.1007/s00213-007-0795-y

[42] Markram K, Rinaldi T, La Mendola D, Sandi C, Markram H (2008) Abnormal fear conditioning and amygdala processing in an animal model of autism. Neuropsychopharmacology 33: 901–912. 17507914 10.1038/sj.npp.1301453

[43] Chiao JY, Iidaka T, Gordon HL, Nogawa J, Bar M, et al (2008) Cultural specificity in amygdala response to fear faces. J Cogn Neurosci 20: 2167–2174. 18457504 10.1162/jocn.2008.20151

[44] McRae K, Gross JJ, Weber J, Robertson ER, Sokol-Hessner P, et al (2012) The development of emotion regulation: an fMRI study of cognitive reappraisal in children, adolescents and young adults. Soc Cogn Affect Neurosci 7: 11–22. 22228751 10.1093/scan/nsr093 PMC3252634

[45] Keightley ML, Chiew KS, Winocur G, Grady CL (2007) Age-related differences in brain activity underlying identification of emotional expressions in faces. Soc Cogn Affect Neurosci 2: 292–302. 18985135 10.1093/scan/nsm024 PMC2566756

[46] Flagstad P, Mork A, Glenthoj BY, van Beek J, Michael-Titus AT, et al (2004) Disruption of neurogenesis on gestational day 17 in the rat causes behavioral changes relevant to positive and negative schizophrenia symptoms and alters amphetamine-induced dopamine release in nucleus accumbens. Neuropsychopharmacology 29: 2052–2064. 15199377 10.1038/sj.npp.1300516

[47] Lin HC, Tseng YC, Mao SC, Chen PS, Gean PW (2011) GABAA receptor endocytosis in the basolateral amygdala is critical to the reinstatement of fear memory measured by fear-potentiated startle. J Neurosci 31: 8851–8861. 21677169 10.1523/JNEUROSCI.0979-11.2011 PMC6622947

[48] Yu SY, Wu DC, Liu L, Ge Y, Wang YT (2008) Role of AMPA receptor trafficking in NMDA receptor-dependent synaptic plasticity in the rat lateral amygdala. J Neurochem 106: 889–899. 18466342 10.1111/j.1471-4159.2008.05461.x

[49] Washburn MS, Moises HC (1992) Electrophysiological and morphological properties of rat basolateral amygdaloid neurons in vitro. J Neurosci 12: 4066–4079. 1403101 10.1523/JNEUROSCI.12-10-04066.1992 PMC6575963

[50] Evans DW, Canavera K, Kleinpeter FL, Maccubbin E, Taga K (2005) The fears, phobias and anxieties of children with autism spectrum disorders and Down syndrome: comparisons with developmentally and chronologically age matched children. Child Psychiatry Hum Dev 36: 3–26. 16049642 10.1007/s10578-004-3619-x

[51] Barad M, Gean PW, Lutz B (2006) The role of the amygdala in the extinction of conditioned fear. Biol Psychiatry 60: 322–328. 16919522 10.1016/j.biopsych.2006.05.029

[52] Hess G, Kuhnt U, Voronin LL (1987) Quantal analysis of paired-pulse facilitation in guinea pig hippocampal slices. Neurosci Lett 77: 187–192. 3601229 10.1016/0304-3940(87)90584-2

[53] Lin HC, Mao SC, Su CL, Gean PW (2010) Alterations of excitatory transmission in the lateral amygdala during expression and extinction of fear memory. Int J Neuropsychopharmacol 13: 335–345. 19775504 10.1017/S1461145709990678

[54] Bellone C, Luscher C (2006) Cocaine triggered AMPA receptor redistribution is reversed in vivo by mGluR-dependent long-term depression. Nat Neurosci 9: 636–641. 16582902 10.1038/nn1682

[55] Clem RL, Barth A (2006) Pathway-specific trafficking of native AMPARs by in vivo experience. Neuron 49: 663–670. 16504942 10.1016/j.neuron.2006.01.019

[56] Du J, Creson TK, Wu LJ, Ren M, Gray NA, et al (2008) The role of hippocampal GluR1 and GluR2 receptors in manic-like behavior. J Neurosci 28: 68–79. 18171924 10.1523/JNEUROSCI.3080-07.2008 PMC2763546

[57] Schneider T, Roman A, Basta-Kaim A, Kubera M, Budziszewska B, et al (2008) Gender-specific behavioral and immunological alterations in an animal model of autism induced by prenatal exposure to valproic acid. Psychoneuroendocrinology 33: 728–740. 18396377 10.1016/j.psyneuen.2008.02.011

[58] Iwasaki-Sekino A, Mano-Otagiri A, Ohata H, Yamauchi N, Shibasaki T (2009) Gender differences in corticotropin and corticosterone secretion and corticotropin-releasing factor mRNA expression in the paraventricular nucleus of the hypothalamus and the central nucleus of the amygdala in response to footshock stress or psychological stress in rats. Psychoneuroendocrinology 34: 226–237. 18849120 10.1016/j.psyneuen.2008.09.003

[59] Kataoka S, Takuma K, Hara Y, Maeda Y, Ago Y, et al (2011) Autism-like behaviours with transient histone hyperacetylation in mice treated prenatally with valproic acid. Int J Neuropsychopharmacol In press. 10.1017/S1461145711001714 22093185

[60] Bachevalier J, Loveland KA (2006) The orbitofrontal-amygdala circuit and self-regulation of social-emotional behavior in autism. Neurosci Biobehav Rev 30: 97–117. 16157377 10.1016/j.neubiorev.2005.07.002

[61] Kleinhans NM, Richards T, Weaver K, Johnson LC, Greenson J, et al (2010) Association between amygdala response to emotional faces and social anxiety in autism spectrum disorders. Neuropsychologia 48: 3665–3670. 20655320 10.1016/j.neuropsychologia.2010.07.022 PMC3426451

[62] Rinaldi T, Kulangara K, Antoniello K, Markram H (2007) Elevated NMDA receptor levels and enhanced postsynaptic long-term potentiation induced by prenatal exposure to valproic acid. Proc Natl Acad Sci U S A 104: 13501–13506. 17675408 10.1073/pnas.0704391104 PMC1948920

[63] Rubenstein JL, Merzenich MM (2003) Model of autism: increased ratio of excitation/inhibition in key neural systems. Genes, Brain, and Behavior 2: 255–267. 10.1034/j.1601-183x.2003.00037.x PMC6748642 14606691

[64] Whalley K (2012) Neurodevelopmental disorders: a fragile synaptic balance. Nat Rev Neurosci 13: 3. 22295280 10.1038/nrn3163

[65] Yizhar O, Fenno LE, Prigge M, Schneider F, Davidson TJ, et al (2011) Neocortical excitation/inhibition balance in information processing and social dysfunction. Nature 477: 171–178. 21796121 10.1038/nature10360 PMC4155501

[66] Walcott EC, Higgins EA, Desai NS (2011) Synaptic and intrinsic balancing during postnatal development in rat pups exposed to valproic acid in utero. J Neurosci 31: 13097–13109. 21917793 10.1523/JNEUROSCI.1341-11.2011 PMC6623264

[67] Marin O (2012) Interneuron dysfunction in psychiatric disorders. Nat Rev Neurosci 13: 107–120. 22251963 10.1038/nrn3155

[68] Nelson ED, Bal M, Kavalali ET, Monteggia LM (2011) Selective impact of MeCP2 and associated histone deacetylases on the dynamics of evoked excitatory neurotransmission. J Neurophysiol 106: 193–201. 21511710 10.1152/jn.00751.2010 PMC3129735

[69] Barrow SL, Constable JR, Clark E, El-Sabeawy F, McAllister AK, et al (2009) Neuroligin1: a cell adhesion molecule that recruits PSD-95 and NMDA receptors by distinct mechanisms during synaptogenesis. Neural Development 4: 17. 19450252 10.1186/1749-8104-4-17 PMC2694798

[70] Craig AM, Kang Y (2007) Neurexin-neuroligin signaling in synapse development. Curr Opin Neurobiol 17: 43–52. 17275284 10.1016/j.conb.2007.01.011 PMC2820508

[71] Scheiffele P, Fan J, Choih J, Fetter R, Serafini T (2000) Neuroligin expressed in nonneuronal cells triggers presynaptic development in contacting axons. Cell 101: 657–669. 10892652 10.1016/s0092-8674(00)80877-6

[72] Jung SY, Kim J, Kwon OB, Jung JH, An K, et al (2010) Input-specific synaptic plasticity in the amygdala is regulated by neuroligin-1 via postsynaptic NMDA receptors. Proc Natl Acad Sci U S A 107: 4710–4715. 20176955 10.1073/pnas.1001084107 PMC2842073

[73] Blundell J, Blaiss CA, Etherton MR, Espinosa F, Tabuchi K, et al (2010) Neuroligin-1 deletion results in impaired spatial memory and increased repetitive behavior. J Neurosci 30: 2115–2129. 20147539 10.1523/JNEUROSCI.4517-09.2010 PMC2824441

[74] Etherton MR, Tabuchi K, Sharma M, Ko J, Sudhof TC (2011) An autism-associated point mutation in the neuroligin cytoplasmic tail selectively impairs AMPA receptor-mediated synaptic transmission in hippocampus. EMBO J 30: 2908–2919. 21642956 10.1038/emboj.2011.182 PMC3160244

[75] Sudhof TC (2008) Neuroligins and neurexins link synaptic function to cognitive disease. Nature 455: 903–911. 18923512 10.1038/nature07456 PMC2673233

